# The Therapeutic Administration of *Lactobacillus brevis* ZG2488 Suppresses Influenza A Virus Replication Through a Viability-Dependent Host Transcriptional Modulation Mechanism

**DOI:** 10.3390/microorganisms14030586

**Published:** 2026-03-05

**Authors:** Mengshan Chen, Yulu Chen, Zhijie Cao, Zhihong Ren, Kun Yue, Jing Yang, Ji Pu, Wenbo Luo, Jianguo Xu

**Affiliations:** 1 School of Medicine, Nankai University, Tianjin 300071, China; 1120210665@mail.nankai.edu.cn; 2 National Key Laboratory of Intelligent Tracking and Forecasting for Infectious Diseases, National Institute for Communicable Disease Control and Prevention, Beijing 102206, China; chenyulu86@163.com (Y.C.); caozhijie@icdc.cn (Z.C.); renzhihong@icdc.cn (Z.R.); yangjing@icdc.cn (J.Y.); puji@icdc.cn (J.P.); luowenbo2021@126.com (W.L.); 3 Research Unite for Unknown Microbe, Institute of Pathogen Biology, Chinese Academy of Medical Sciences & Peking Union Medical College, Beijing 100730, China; kunyue95@163.com; 4 Research Center for Reverse Microbial Etiology, Workstation of Academician, Shanxi Medical University, Taiyuan 030001, China

**Keywords:** therapeutic probiotic, *Lactobacillus brevis*, influenza A virus, host–pathogen interaction, antiviral mechanism, untargeted metabolomics

## Abstract

Influenza A virus (IAV) remains a major global threat, highlighting the need for host-targeted antiviral strategies. While some probiotics offer prophylactic protection, their therapeutic potential post-infection is poorly understood. Here, we investigated human-derived *Lactobacillus brevis* ZG2488 for its antiviral potential against IAV. Strikingly, a more pronounced reduction in viral titer was observed when live bacteria were administered therapeutically post-infection, compared to preventive pretreatment. Transcriptomic analysis suggested that the therapeutic effect of viable bacteria was associated with a modulated host response, including the downregulation of specific host factors implicated in viral replication (e.g., *KPNA2*, *NUP98*, *EIF2S1*) and a delayed interferon-beta (IFNB1) induction. In contrast, preventive effects appeared to be mediated by heat-stable components. These findings highlight a viability-dependent mode of action for *L. brevis* ZG2488 and contribute to the growing evidence that certain probiotics may exert antiviral effects through targeted host modulation rather than solely through broad immune activation.

## 1. Introduction

Influenza A virus (IAV) poses a major and ongoing threat to global public health due to its high mutation rate and frequent epidemics [1,2,3]. Although vaccination and antiviral drugs remain the mainstream countermeasures, antigenic drift and shift reduce vaccine efficacy, highlighting the urgent need for novel antiviral strategies [4,5,6]. Even though there is a lot of evidence to support the application of probiotics in the prevention of respiratory infections (its good safety and wide range of immunomodulatory properties have attracted much attention), the reported mechanism of action is usually limited to non-specific immune activation or barrier enhancement [7,8]. In contrast, there is still a huge knowledge gap in the therapeutic application of probiotics after viral infection and their exact mechanism of action. We assume that certain specific probiotics can go beyond this traditional paradigm by actively reprogramming host cells to establish an antiviral state as an effector of host-oriented therapy.

A large number of studies have shown that probiotics such as Lactobacillus and Bifidobacterium can effectively prevent viral infections by enhancing mucosal barriers, competitively rejecting pathogens and activating innate immunity [9,10,11,12]. However, most studies focus on prevention, and little is known about the therapeutic effect after infection [13,14]. In addition, its potential mechanisms are usually summarized as “immune enhancement”, without an in-depth exploration of more complex molecular mechanisms—especially those involving the direct regulation of host factors required for viral replication [15,16,17].

*Lactobacillus brevis* (referred to as *L. brevis*) is a common lactic acid bacterium that exists in human intestines and fermented foods. Some strains have been reported to have immunomodulatory potential [18,19,20]. Building upon recent evidence that probiotic strains and their derivatives—such as extracellular vesicles or inactivated forms—can modulate host metabolic reprogramming, autophagy, or cellular vitality [21,22,23], we sought to explore whether ZG2488 offers protection through mechanisms distinct from conventional host defense enhancement [24]. In this study, we aimed to investigate the antiviral activity of the human-derived strain *L. brevis* ZG2488 against IAV and characterize its impact on host cellular pathways by employing an integrated approach involving in vitro prevention and treatment models, transcriptomic profiling, and in vivo murine experiments. Our observations suggest that ZG2488 treatment is associated with the systemic downregulation of host factors required for IAV replication, resulting in a host transcriptional modulation phenotype. This work provides a preliminary framework for understanding host-centric probiotic interventions and offers a potential direction for developing microbiota-based antiviral strategies.

## 2. Methods

### 2.1. Cells, Viruses, and Bacterial Strains

The human lung epithelial cell line A549 was obtained from the American Type Culture Collection (ATCC, Manassas, VA, USA) and cultured in Dulbecco’s Modified Eagle Medium (DMEM, Gibco, Grand Island, NY, USA) supplemented with 10% fetal bovine serum (FBS, Gibco, Grand Island, NY, USA) and 1% penicillin–streptomycin at 37 °C in a humidified incubator with 5% CO_2_. The mouse-adapted influenza A virus strain A/Puerto Rico/8/1934 (H1N1, PR8) was provided by the Chinese National Influenza Center (CNIC, Beijing, China) and propagated in embryonated chicken eggs. Viral titers were determined in Madin–Darby canine kidney (MDCK) cells (ATCC, Manassas, VA, USA) using the 50% tissue culture infectious dose (TCID_50_) assay and plaque assay.

*L. brevis* ZG2488, previously isolated from the gut microbiota of a healthy individual [20,25], was cultured anaerobically in de Man–Rogosa–Sharpe (MRS) broth (Oxoid, Basingstoke, UK) at 37 °C to the mid-logarithmic growth phase for experimental use. Heat-killed *L. brevis* ZG2488 (referred to as HK-*L. brevis* ZG2488) was prepared by harvesting bacterial cells, washing them three times with phosphate-buffered saline (PBS, Oxoid, Basingstoke, UK), resuspending them in PBS, and heating at 70 °C for 30 min in a water bath. The complete loss of viability was confirmed by plate counting [21,26,27,28].

### 2.2. In Vitro Antiviral Assays

To evaluate antiviral activity, both preventive and therapeutic models were established.

Preventive model: A549 cells were seeded into 12-well plates (Corning, Corning, NY, USA) at an appropriate density and incubated for 24 h. Cells were then treated with live *L. brevis* ZG2488 (multiplicity of infection, MOI = 100) or HK-*L. brevis* ZG2488 (MOI = 100) in maintenance medium (DMEM with 2% FBS) for either 6 h or 24 h. After pretreatment, cells were washed three times with PBS to remove bacteria, followed by infection with PR8 virus at MOI = 1 for 1 h. The inoculum was removed, cells were washed, and fresh maintenance medium was added. Samples were collected at designated time points. Therapeutic model: A549 cells were first infected with PR8 virus (MOI = 1) for 1 h. After viral adsorption, the inoculum was removed and replaced with maintenance medium containing live *L. brevis* ZG2488 (MOI = 100) or HK-*L. brevis* ZG2488. Samples were collected at 6 h and 24 h post-infection. Control groups included virus-only infection and uninfected cell controls. Collected cell samples were used for viral load quantification by quantitative real-time PCR (qPCR) or RNA extraction.

### 2.3. Transcriptome Sequencing and Analysis

For the therapeutic model, A549 cells were harvested at 24 h post-infection, with three biological replicates per group. RNA sequencing was performed on the Illumina NovaSeq 6000 platform. After quality control and alignment, differential gene expression analysis was conducted using the DESeq2 package(v1.42.0) in the R environment (version 4.5.1). To correct for false positives due to multiple testing, *p*-values were adjusted using the Benjamini–Hochberg method to generate false discovery rates (FDRs). Genes with adjusted *p* < 0.05 and |log_2_ fold change| > 1 were defined as significantly differentially expressed. Gene set enrichment analysis (GSEA) was performed using the clusterProfiler package (v4.10.0) in R, with the Hallmark gene sets as the reference [29]. Enriched pathways with FDR < 0.05 were considered statistically significant.

### 2.4. Mouse Influenza Model

Female BALB/c mice (6–8 weeks old) were randomly assigned to preventive, therapeutic, or virus control groups (*n* = 6 per group). Preventive group: Mice were orally gavaged daily with 200 μL of *L. brevis* ZG2488 suspension (~1 × 10^9^ CFU) or 200 μL PBS for three consecutive days prior to intranasal inoculation. Following anesthesia, mice underwent intranasal infection with 450 PFU of the PR8 strain on the designated infection day (Day 0). Therapeutic group: Mice were gavaged with the same bacterial suspension or PBS immediately after viral infection and continued daily thereafter. At 3 days post-infection, mice were sacrificed, and lung tissues were collected. Lung tissues were homogenized, and RNA was extracted from the clarified homogenate supernatants using TRIzol™ reagent (Cat#15596018, Invitrogen, Carlsbad, CA, USA) for subsequent qPCR analysis. All animal care and experimental procedures were reviewed and approved by the Laboratory Animal Welfare and Ethics Committee of the National Institute for Communicable Disease Control and Prevention, Chinese Center for Disease Prevention and Control (Approval number: 2023-025, approved 5 June 2023, Beijing, China).

### 2.5. Viral Load Quantification by qPCR

Viral titers were determined by homogenizing the entire lung or collected cell samples in 1 mL of Trizol™ reagent (Cat#15596018, Invitrogen, Carlsbad, CA, USA). Total RNA was then extracted following the manufacturer’s protocol. Subsequently, the RNA was diluted to a concentration of 1 μg/μL. qPCR was performed using an influenza A virus detection kit (Cat#CN104C-100, Jiangsu Uninovo Biological Technology Co., Ltd., Zhenjiang, China) on an Applied Biosystems 7500 system (Thermo Fisher Scientific, Waltham, MA, USA) to measure the viral load. The results were expressed as the mean ± standard deviation (SD) from at least three independent experiments. Statistical comparisons were performed using Student’s *t*-test, and *p* < 0.05 was considered statistically significant.

### 2.6. Whole-Genome Sequencing and Bioinformatic Analysis

The genomic DNA of *L. brevis* ZG2488 was extracted and sequenced on the Illumina NovaSeq 6000 platform (Illumina, San Diego, CA, USA). After quality control and assembly, the genome was functionally annotated using multiple databases: the NCBI non-redundant protein (NR) database for general protein function prediction, the Kyoto Encyclopedia of Genes and Genomes (KEGG) for metabolic pathway and functional classification, the Carbohydrate-Active enZymes (CAZy, via dbCAN2) database for carbohydrate-active enzyme annotation, the Pfam database for protein domain identification, the Transporter Classification Database (TCDB) for transporter proteins, the Virulence Factors Database (VFDB) for virulence-related genes, and the Comprehensive Antibiotic Resistance Database (CARD) for antimicrobial resistance determinants [30,31,32]. This integrative annotation approach provided a comprehensive view of the metabolic potential, safety, and functional characteristics of *L. brevis* ZG2488 [33].The 16S rRNA gene sequence of *L. brevis* ZG2488 has been deposited in GenBank under the accession number PQ849070 and designated as *L. brevis* ZG2488 PQ849070.

### 2.7. Untargeted Metabolomic Analysis

To characterize the secretome of live *L. brevis* ZG2488, an untargeted metabolomic analysis was performed on its cell-free supernatant. The bacteria were cultured in MRS broth to the mid-logarithmic phase. The supernatant was collected by centrifugation (12,000× *g*, 10 min), followed by filtration through a 0.22 µm membrane (Merck Millipore, Burlington, MA, USA) to obtain a sterile filtrate. An equal volume of sterile MRS medium served as the control. All samples were immediately frozen at −80 °C.

Metabolite extraction was performed using a methanol–acetonitrile–water system. Liquid chromatography–tandem mass spectrometry (LC-MS/MS) analysis was conducted on a system equipped with an electrospray ionization source, operating in both positive and negative ion modes with data-dependent acquisition.

Raw data were processed using Progenesis QI software (v3.1) for peak picking, alignment, and integration. Metabolites were identified by searching against the HMDB, Metlin, and in-house standard databases and annotated using the KEGG database. After filtering out features with >20% missing values within any group and those with a relative standard deviation (RSD) >30% in quality control samples, the data were subjected to missing value imputation, sum normalization, and log10 transformation to generate the final data matrix.

Statistical analysis included principal component analysis to assess inter-group separation trends and data quality. Differential metabolites were screened by combining the variable importance in projection (VIP > 1) from an orthogonal partial least squares discriminant analysis (OPLS-DA) model with Student’s *t*-test (*p* < 0.05). An enrichment analysis of differential metabolites in KEGG pathways was performed using a hypergeometric test, with a false discovery rate (FDR) < 0.05 considered significant.

### 2.8. Data Analysis

All in vitro experiments were performed with at least three independent biological replicates. In vivo experiments used a minimum of five mice per group. Data were expressed as the mean ± SD. Statistical analyses and graphing were performed using GraphPad Prism software (version 10.6.1,GraphPad Software, San Diego, CA, USA). Student’s *t*-test was applied for two-group comparisons. For multiple group comparisons involving different time points, linear mixed-effects models (LMMs) were used to account for both fixed and random effects. *p* < 0.05 was considered statistically significant. Significance levels are denoted as *p* < 0.05 (*), *p* < 0.01 (**), and *p* < 0.001 (***).

## 3. Results

### 3.1. Differential Antiviral Effects of Preventive and Therapeutic Interventions In Vitro

**Figure 1:**
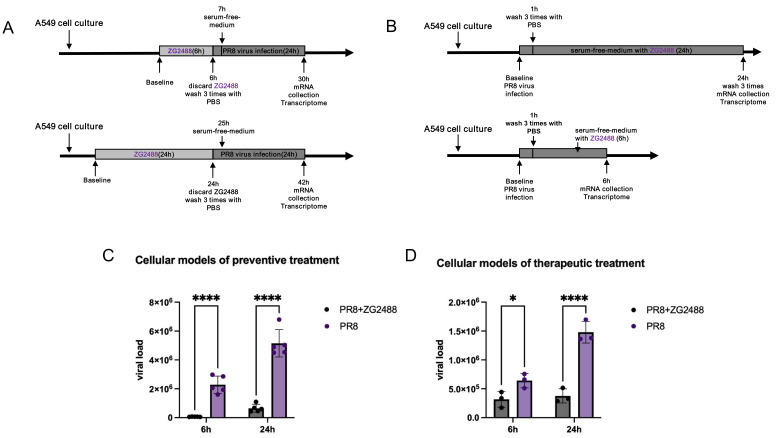
Antiviral activity of *Lactobacillus brevis* ZG2488 in preventive and therapeutic A549 cell models. (**A**,**B**) Experimental schemes for preventive (**A**) and therapeutic (**B**) treatments. A549 cells were either pretreated with *L. brevis* ZG2488 before PR8 infection or treated after infection. (**C**,**D**) qPCR analysis of viral load showing significant inhibition by *L. brevis* ZG2488 compared with PR8-only controls (* *p* < 0.05, **** *p* < 0.0001). Preventive effects appeared earlier, while therapeutic effects strengthened over time. Data are shown as mean ± SEM (*n* = 3–6); significance was determined by two-tailed unpaired *t*-test.

To systematically evaluate the antiviral potential of *L. brevis* ZG2488, we established both preventive and therapeutic intervention models in A549 cells (Figure 1A,B).

At 24 h post-infection, the qPCR quantification of viral load revealed a highly significant reduction in viral load compared with the virus control group (PR8-V) for both pretreatment durations (*p* < 0.001). Specifically, the 6 h pretreatment showed the most pronounced effect, with an average 30-fold reduction in viral load, whereas an approximately 24 h pretreatment resulted in a more modest 8-fold reduction (Figure 1C). These results suggest that *L. brevis* ZG2488 is associated with the induction of an antiviral state in host cells, which may reflect a “pre-activation” mechanism that weakens over time.

In the treatment model, the cells were first infected with PR8 virus (MOI = 1) for 1 h, and then *L. brevis* ZG2488 (1 × 10^8^ CFU/mL) was added to the maintenance medium. At 6 h post-infection, a slight difference in viral load was observed between the treatment and virus control groups (*p* < 0.05, *n* = 6). However, by 24 h post-infection, *L. brevis* ZG2488 treatment led to a significant reduction in viral load, averaging an approximately 42-fold decrease compared with controls (*p* < 0.0001, *n* = 6) (Figure 1D).

Collectively, these results indicate that *L. brevis* ZG2488 exerts dual antiviral activities in vitro: it can rapidly prime host cells into a defensive state prior to viral exposure, and it can also suppress viral replication after infection has been established. These effects display a temporal dependence, with preventive efficacy peaking early and therapeutic efficacy strengthening over time.

### 3.2. Transcriptomic Analysis Reveals Downregulation of Host Factors Under Therapeutic Intervention

To investigate the molecular basis of the therapeutic effect, we performed RNA sequencing (RNA-seq) on cell samples collected at 6 h post-infection (6 hpi) and 24 h post-infection (24 hpi) in the therapeutic model.

**Figure 2:**
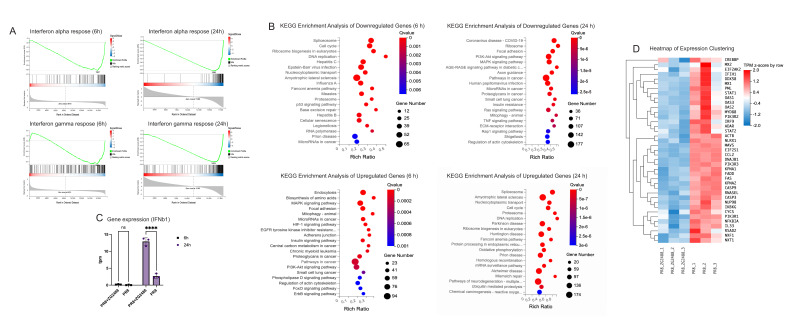
Transcriptomic analysis of A549 cells under therapeutic treatment with *Lactobacillus brevis* ZG2488. (**A**) GSEA showing suppression of interferon-α and interferon-γ response pathways at 6 h and 24 h post-infection. (**B**) KEGG enrichment analysis of up- and downregulated genes, highlighting inhibition of influenza A, RNA polymerase, and nucleocytoplasmic transport pathways. (**C**) IFNB1 expression (TPM) showed no change at 6 h and strong upregulation at 24 h (**** *p* < 0.0001, ns = not significant). (**D**) Heatmap of representative host genes showing reduced expression of KPNA2, NUP98, EIF2S1, CREBBP, MX1, and OAS2 after ZG2488 treatment.

Gene set enrichment analysis (GSEA) revealed that, compared with the virus control group (PR8-V), *L. brevis* ZG2488 treatment was associated with the downregulation of the interferon-α response and interferon-γ response pathways at both 6 hpi and 24 hpi (6 hpi: NES < −2.0, FDR < 0.001; 24 hpi: NES < −1.8, FDR < 0.01). This suggests that *L. brevis* ZG2488 may modulate the inflammatory signaling pathway associated with viral infection (Figure 2A).

KEGG enrichment analysis further indicates that the downregulated genes in the *L. brevis* ZG2488 treatment group are significantly enriched in host pathways associated with virus replication, including influenza A virus, RNA polymerase and nucleal transport (Figure 2B). A heatmap analysis of the key host genes in the influenza A virus pathway (Figure 2D) shows that nuclear transport factors (such as KPNA2, NUP98), transcription and translation regulatory factors (such as EIF2S1, CREBBP) and interferon-stimulating genes (such as MX1, OAS2) all showed suppression.

Notably, the expression of type I interferon β (IFNB1) in the RNA-seq data set reveals a timing regulation. Quantitative analysis per million transcripts (TPM) showed that there was no significant difference in IFNB1 expression between the *L. brevis* ZG2488 treatment group and the viral control group 6 h after infection (*p* > 0.05). However, by 24 h after infection, the expression level of IFNB1 in the *L. brevis* ZG2488 treatment group was significantly higher than that of the control group (Figure 2C).

In summary, transcriptomic analysis reveals that ZG2488 treatment induces a temporally modulated interferon response. At 6 hpi, both interferon-α/γ response pathways and key interferon-stimulated genes (ISGs) like MX1 and OAS2 are downregulated, concurrent with the suppression of proviral host factors. Notably, this early phase is characterized by the absence of a significant IFNB1 induction. By 24 hpi, however, a delayed yet pronounced upregulation of IFNB1 is observed. This pattern suggests that early viral replication is potentially constrained via host factor downregulation, which may subsequently permit a more regulated and potentially effective type I interferon response at a later time point, differing from a classical early interferon burst.

### 3.3. Functional Validation Demonstrates Distinct Mechanisms for Preventive and Therapeutic Effects

To validate the underlying mechanisms of the two modes of action, we compared the effects of live *L. brevis* ZG2488 and heat-killed *L. brevis* ZG2488 (HK-*L. brevis* ZG2488, treated at 80 °C for 30 min) in both the preventive and therapeutic models.

**Figure 3:**
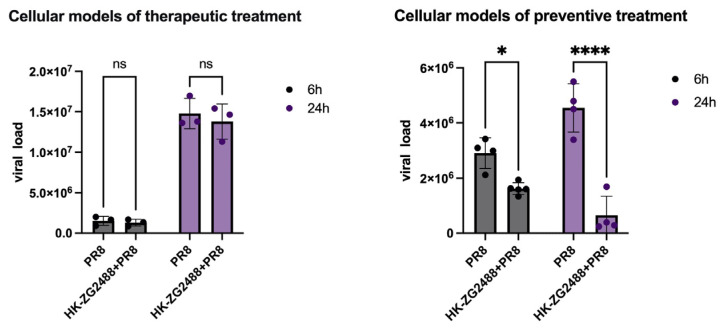
Functional verification reveals the different mechanisms of the preventive and therapeutic antiviral effects of *L. brevis* ZG2488. We established (**left**) therapeutic (**left**) and (**right**) preventive treatment cell models to evaluate the antiviral activity of heat-killed *L. brevis* ZG2488 (HK-ZG2488). The viral load is quantified 6 h (black) and 24 h (purple) after infection. In the treatment model, HK-ZG2488 showed no detectable antiviral effect compared with the PR8 control group (ns, *p* > 0.05). On the contrary, in the prevention model, HK-ZG2488 significantly inhibited viral replication (* *p* < 0.05, **** *p* < 0.0001, ns > 0.05). The data is represented by the average ± standard error (SEM) (n = 4). A single-factor analysis of variance (ANOVA) and Tukey post-test are used to determine statistical significance.

In the preventive model, treatment with HK-*L. brevis* ZG2488 (10^8^ CFU/mL) still resulted in a significant reduction in viral load compared with the virus control group (Figure 3). Notably, this effect was statistically indistinguishable from that of live bacteria (*p* > 0.05) (Figure 1C). These findings indicate that preventive protection is primarily mediated by heat-stable bacterial structural components, such as surface layer proteins and capsular polysaccharides, which may physically block viral entry by competitively binding to host cell surface receptors.

In contrast, in the therapeutic model, HK-*L. brevis* ZG2488 treatment failed to significantly reduce viral load (*p* > 0.05, *n* = 4), with levels comparable to the virus control group (Figure 3). On the contrary, the living *L. brevis* ZG2488 still maintains strong viral clearance activity (*p* < 0.0001) (Figure 1D). This indicated that the therapeutic effect is dependent on the metabolic activity of live bacteria and/or the dynamic interaction with the host and cannot be generated by the structural components of the bacteria alone.

In summary, these results point toward two antiviral profiles of *L. brevis* ZG2488: the preventive protective effect is mediated by heat-resistant structural components, such as surface layer (S-layer) proteins, capsular polysaccharides, or adhesins, which may physically block viral entry by competitively binding to host cell surface receptors; the therapeutic removal effect depends strictly on live bacteria, which may occur through the active regulation of the host group. It is realized by expression and immune response.

### 3.4. In Vivo Verification: Oral Intervention Can Reduce the Viral Load in the Lungs of Mice

**Figure 4:**
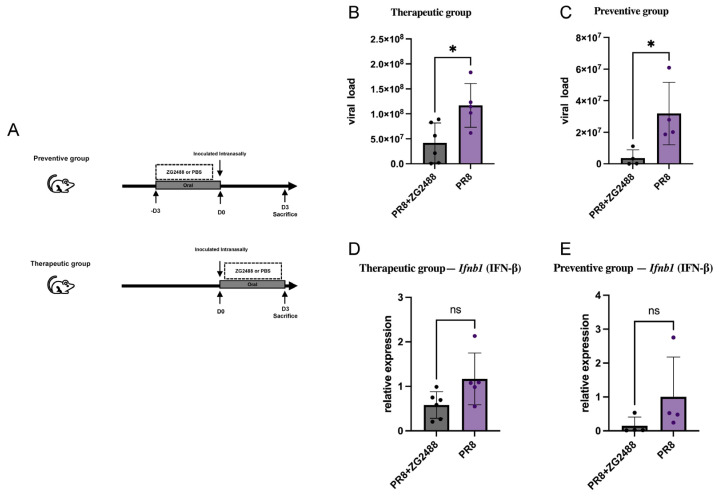
In vivo validation of oral *Lactobacillus brevis* ZG2488 intervention confers non-inflammatory protection against influenza infection. (**A**) Experimental design of preventive (top) and therapeutic (bottom) models in BALB/c mice. *L. brevis* ZG2488 (1 × 10^9^ CFU) or PBS was administered daily by oral gavage. Preventive treatment started three days before intranasal PR8 challenge, while therapeutic treatment began post-infection. (**B**,**C**) Lung viral load measured by qPCR on Day 3 post-infection. Both preventive and therapeutic ZG2488 groups showed significantly reduced viral titers compared with PR8 controls (* *p* < 0.05). (**D**,**E**) Relative expression of Ifnb1 (IFN-β) lmRNA in lung tissues of preventive (**D**) and therapeutic (**E**) models showed downward but non-significant trend versus control (ns > 0.05). Data are presented as mean ± SEM (*n* = 4). Statistical significance was determined using one-way ANOVA with Tukey’s post hoc test.

To evaluate the in vivo efficacy of *L. brevis* ZG2488, we performed oral administration experiments using a BALB/c mouse influenza model (Figure 4A). qPCR quantification revealed that both the preventive and therapeutic groups exhibited a marked reduction in lung viral load compared with the infection control group (Figure 4B,C), indicating that the antiviral effects observed in vitro were consistent with those in a physiological setting.

An analysis of interferon-related genes in lung tissues showed that the mRNA levels of interferon-β (Ifnb1) and interferon-λ (Ifnl2/3) in *L. brevis* ZG2488-treated mice showed a downward trend compared with the infection control group, although the differences did not reach statistical significance (Figure 4D,E). This pattern is consistent with the in vitro transcriptomic data, suggesting that the antiviral effect of *L. brevis* ZG2488 is unlikely to rely on a classical type I/III interferon burst and may instead involve alternative, less inflammatory host regulatory pathways.

Together with the in vitro evidence of host factor downregulation, these findings are consistent with the hypothesis that *L. brevis* ZG2488 mediates a form of “non-inflammatory immune modulation”, potentially via systemic regulation through the gut–lung axis. By enabling efficient viral clearance while maintaining regulated interferon-driven inflammation, *L. brevis* ZG2488 demonstrates both biosafety and therapeutic promise as an orally administered probiotic candidate.

### 3.5. Genome Annotation Reveals the Molecular Basis for Antiviral Phenotypes

To elucidate the genetic foundation underlying the observed antiviral functions of *L. brevis* ZG2488, we conducted a comprehensive annotation of its draft genome. The analysis reveals the multifaceted genetic mechanisms of the strain, which provide a potential genetic basis for its prevention and treatment potential (Table 1).

It is worth noting that the KEGG pathway analysis identified the complete metabolic pathways of short-chain fatty acids (SCFAs), including key genes involved in the metabolism of pyruvate (23 genes), propionic acid (19 genes) and butyric acid (11 genes). This indicates that metabolites such as butyric acid may potentially participate in host immune regulation as signaling molecules. In addition, NR annotation reveals a complete group sensing system, which contains six histidine kinase and four reaction regulatory genes, indicating that the strain can dynamically regulate the synthesis of effect molecules according to environmental signals.

CAZy annotation and Pfam annotation support the genetic basis of the “space blocking” effect. CAZy annotation revealed 45 glycosyltransferase (GT) genes and 38 glycoside hydrolase (GH) genes; Pfam annotation identified 5 bacterial adhesion genes. Notably, we identified a gene encoding a putative colicin-like translocation domain, suggesting a potential mechanism for the delivery of effector molecules to host cells, which aligns with the observed viability-dependent “host transcriptional modulation”. TCDB annotation supports the ability of the strain to secrete these molecules, and the annotation predicts 35 ABC transporters. Finally, the pathogenicity analysis confirmed that the strain does not carry known virulence genes or antibiotic resistance genes, highlighting its safety.

In a word, genome annotation provides a potential molecular explanation for the observed antiviral phenotypes, revealing that *L. brevis* ZG2488 has a genetic toolkit for prevention (through structural components) and treatment phenomena (through active signal conduction and potential host regulation) [34,35].

**Table 1:** Summary of genomic features of *L. brevis* ZG2488 underlying its antiviral phenotypes.

Annotation Database	Key Findings	Gene Count	Representative Gene/Pathway	Proposed Functional Significance
KEGG	Short-chain fatty acid (SCFA) metabolic pathways	53 (total)	Pyruvate/Propionate/Butyrate metabolism	Production of immunomodulatory metabolites (e.g., butyrate) [36]
NR	Two-component system (quorum sensing)	10 (total)	Histidine kinase/Response regulator	Sensing environment and dynamically regulating effector synthesis [37]
CAZy	Glycosyltransferases (GTs)	45	GT2, GT4, GT8, GT26, GT28, GT30, GT51 families	Synthesis of surface polysaccharides for spatial blocking [38,39]
	Glycoside hydrolases (GHs)	38	GH23, GH25, GH73 families	Peptidoglycan remodeling and release of immunostimulatory fragments [40,41]
Pfam	Bacterial adhesins	5	Bacterial adhesin superfamily	Host cell attachment, enhancing physical barrier effect [11,42]
	Putative colicin-like bacteriocin translocation domain	1	Colicin-like bacteriocin domain	Encodes putative domain; its potential role in effector molecule delivery remains genomic prediction that warrants future experimental validation [43,44]
TCDB	ABC transporters	35	ABC transporter proteins	Efficient secretion of metabolites and effector molecules [45,46]

### 3.6. Metabolomic Profiling Uncovers Specific Metabolic State Adaptation in Live ZG2488

To molecularly decipher the chemical basis of the therapeutic effect of live *L. brevis* ZG2488, untargeted metabolomic analysis was performed on its cell-free supernatant. Compared to the sterile medium control, the live bacteria supernatant displayed a metabolic signature, defined by a selective enrichment in a set of key bioactive molecules and concomitant broad shifts in metabolic pathway activity.

**Figure 5:**
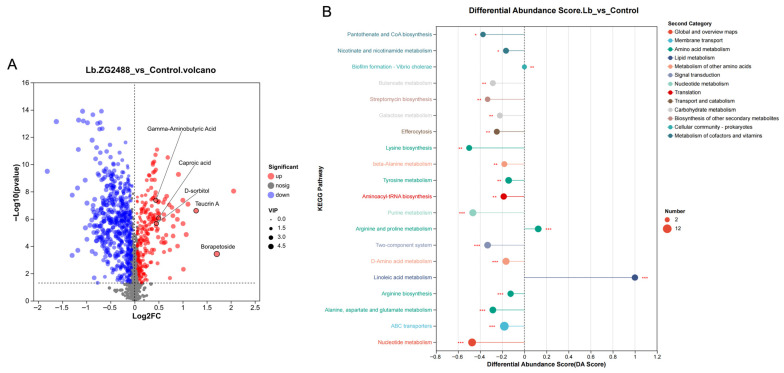
Untargeted metabolomic profiling reveals a metabolic state adaptation in live *Lactobacillus brevis* ZG2488. (**A**) A volcano plot showing differential metabolite abundance between the cell-free supernatant of live *L. brevis* ZG2488 and sterile medium control. The x-axis represents the log2 fold change (Log2FC), and the y-axis represents the −log10 *p*-value. Red dots indicate significantly upregulated metabolites, blue dots indicate significantly downregulated metabolites, and gray dots represent metabolites with no significant difference. Dot size corresponds to the variable importance in projection (VIP) score. Key metabolites, such as Borapetoside, Teucrin A, caproic acid, γ-aminobutyric acid (GABA), and D-sorbitol, are highlighted, showing specific enrichment in the live bacteria supernatant. (**B**) A KEGG pathway enrichment analysis of the differential metabolites based on their differential abundance scores (DA scores). The x-axis shows the DA score, and the y-axis represents the significantly enriched KEGG pathways. The size of the dots reflects the number of metabolites mapped to each pathway, while the color indicates the functional category of the pathway. The dashed vertical line represents DA score = 0 (* *p* < 0.05, ** *p* < 0.001, *** *p* < 0.0001).

The live bacteria supernatant is specifically enriched with structurally unique and functionally defined metabolites (Figure 5A). Differential analysis indicated that the levels of multiple structurally complex microbial secondary metabolites were significantly elevated in the live bacteria supernatant, most prominently Borapetoside (Log2FC = 1.71) and Teucrin A (Log2FC = 1.28). The enrichment in these compounds provides direct evidence of the strain’s unique biosynthetic capacity. Concurrently, we identified the presence of the short-chain fatty acid caproic acid (Log2FC = 0.50), consistent with its annotated metabolic potential in the genome. Furthermore, the immunomodulatory molecule γ-aminobutyric acid (GABA) and the sugar alcohol D-sorbitol, known for its antioxidant properties, were also significantly accumulated in the live bacteria supernatant.

The metabolic pathway enrichment pattern reveals a systemic adjustment of the metabolic state. The KEGG pathway enrichment analysis of the differential metabolites showed their significant aggregation in pathways such as ABC transporters, two-component systems, quorum sensing, and Butanoate metabolism (Figure 5B). A key pattern was observed upon closer inspection: among the top 20 significantly enriched pathways, the differential abundance scores (DA score) for the associated metabolite sets were negative (DA score < 0) for all pathways except Linoleic acid metabolism and Arginine and proline metabolism. This prevalent downward trend, combined with the sharp decrease in the levels of various dipeptide nutrients (e.g., Phe-Ser) in the supernatant, collectively suggests that live ZG2488 undergoes a metabolic state adaptation. This adaptation may involve potential coordination via its quorum sensing and two-component systems to reduce the intensity of background metabolic activity while channeling metabolic resources toward the synthesis and secretion of a specific set of effector molecules.

## 4. Discussion

This study utilized an integrated approach involving in vitro experiments, in vivo mouse models, and multi-omics analyses to evaluate the anti-influenza potential of *Lactobacillus brevis* ZG2488. The findings contribute to our understanding of probiotic modes of action, exploring their role from traditional immune enhancers as potential host-targeted modulators [7,8].

An observation from our study is that *L. brevis* ZG2488 exhibits a distinct time-dependent modality, which can be described as interferon temporal reshaping. Unlike some antiviral strategies that rely on an early, robust interferon response, *L. brevis* ZG2488 was associated with the early downregulated key host factors required for viral replication, such as KPNA2, NUP98, and EIF2S1 [47,48]. This early modulation could potentially limit viral amplification at its source. This containment of the viral lifecycle may alleviate the burden of viral antagonist proteins (e.g., NS1) [49,50], enabling the host to mount a delayed yet more controlled interferon response at a later stage (24 h). This “first suppress, then boost” pattern offers a plausible explanation for the balanced efficacy and low inflammation phenotype observed in our models. This response—characterized by the early downregulation of interferon-related pathways and ISGs (e.g., MX1, OAS2) followed by delayed IFNB1 induction—differs from broad interferon suppression and helps clarify the observed high efficacy with low inflammation.

Multi-omics data are consistent with these observed phenotypes. Genomic annotation identified pathways for SCFA biosynthesis, abundant ABC transporters, and a quorum-sensing system [37]. Metabolomic analysis detected the secretion of caproic acid, an SCFA, and delineated a metabolic state of live *L. brevis* ZG2488, characterized by the concurrent downregulation of basal metabolic pathway activity and upregulation in the secretion of specific effector molecules (e.g., caproic acid, the complex natural product Borapetoside, and the immunomodulatory molecule GABA). This adaptation suggests a potential reallocation of resources toward the synthesis of signaling molecules targeted at the host. This process is associated with bacterial viability, providing a clue for why heat-killed bacteria lose therapeutic function while retaining a prophylactic effect [51,52]. Notably, this viability-dependent secretory phenotype aligns with the a putative colicin-like translocation domain identified in the genome. This genomic feature offers an intriguing hypothetical model for how effector molecules might be delivered to the host interface. It is crucial to emphasize that the function of this domain and its role in delivery remain entirely a genomic prediction. The direct functional evidence supporting active secretion in this study comes from the metabolomic analysis detailed above, which definitively shows that live bacteria secrete a unique set of bioactive molecules. The metabolic evidence, together with the viability-dependent therapeutic phenotype, supports our proposed “active secretion” framework. The predicted transport domain, therefore, represents a molecular candidate that requires future experimental validation (e.g., via gene deletion or protein interaction studies).

We acknowledge that this study has several limitations. First, while our untargeted metabolomic data identified the secretion of molecules such as caproic acid, GABA, and Borapetoside, their individual causal contributions and intracellular targets remain to be fully elucidated. The potential role of host factors (e.g., *KPNA2*, *NUP98*) suggested by transcriptomic analysis could not be formally tested via genetic manipulation in this study due to technical constraints, and this warrants further investigation in future studies. Second, the downstream signaling pathways and the regulatory logic linking bacterial quorum sensing to the observed “metabolic state adaptation” are not yet fully defined and require further investigation. Third, A549 cells and mouse models cannot fully replicate the cellular complexity and immune microenvironment of the human lung. The precise cellular targets of *L. brevis* ZG2488 or its secreted factors (e.g., alveolar epithelial cells, macrophages) remain to be determined. Advanced models such as co-culture systems, lung organoids, or cell-type-specific knockout mice will be crucial to address these questions. Also, the in vivo experiments in this study were conducted over a short observation period (3 days), which was sufficient to assess early viral replication but insufficient to reliably evaluate broader clinical outcomes such as body weight loss, survival, or lung histopathology. Future studies employing extended infection models will be necessary to comprehensively assess therapeutic efficacy across multiple disease-relevant phenotypes. Finally, translational considerations must be addressed. The bacterial concentration and exposure used in vitro and in mice may not directly reflect achievable colonization levels or effective doses in humans. Therefore, systematic dose–response studies and early-phase clinical trials will be essential to translate these preliminary findings of *L. brevis* ZG2488.

## 5. Conclusions

This study provides in vitro and in vivo evidence that the human-derived strain *Lactobacillus brevis* ZG2488 exhibits antiviral activity against IAV. Its modes of antiviral effects are mode-dependent: preventive protection can be mediated by heat-stable bacterial components, whereas therapeutic efficacy strictly requires bacterial viability.

Transcriptomic analysis suggests that the therapeutic effect may involve the downregulation of specific host factors (e.g., *KPNA2*, *NUP98*) beneficial for IAV replication, coupled with a modulated interferon response timeline. Genomic and metabolomic analyses are consistent with this viability-dependent phenotype, revealing genetic potential.

In summary, our results detail the antiviral profile of *L. brevis* ZG2488 and point toward a viability-dependent host-targeted mechanism that warrants further validation.

## Data Availability

The data presented in this study are openly available in A Dual-Phase Antiviral Mechanism of Lactobacillus brevis ZG2488 at https://doi.org/10.6084/m9.figshare.30742544 (accessed on 30 November 2025).

## Abbreviations

The following abbreviations are used in this manuscript:

A549	A549 Human Lung Epithelial Cell Line
ABC transporters	ATP-Binding Cassette Transporters
ATCC	American Type Culture Collection
CARD	Comprehensive Antibiotic Resistance Database
CAZy	Carbohydrate-Active enZymes Database
CFU	Colony-Forming Unit
DMEM	Dulbecco’s Modified Eagle Medium
FBS	Fetal Bovine Serum
FDR	False Discovery Rate
GABA	γ-Aminobutyric Acid
GSEA	Gene Set Enrichment Analysis
GT	Glycosyltransferase
GH	Glycoside Hydrolase
H1N1 (PR8)	A/Puerto Rico/8/1934 (H1N1)
HK-*L. brevis*	Heat-Killed *L. brevis*
IAV	Influenza A Virus
Ifnb1	Interferon Beta 1 Gene
Ifnl2/3	Interferon Lambda 2/3 Gene
IFN-α/β/γ	Interferon-Alpha/Beta/Gamma
ISGs	Interferon-Stimulated Genes
KEGG	Kyoto Encyclopedia of Genes and Genomes
MDCK	Madin–Darby Canine Kidney Cells
MOI	Multiplicity of Infection
MRS	de Man, Rogosa and Sharpe Medium
NES	Normalized Enrichment Score
NR	Non-Redundant Protein Database
PBS	Phosphate-Buffered Saline
Pfam	Protein Family Database
qPCR	Quantitative Real-Time PCR
RNA-seq	RNA Sequencing
SCFAs	Short-Chain Fatty Acids
TCID_50_	50% Tissue Culture Infectious Dose
TCDB	Transporter Classification Database
TPM	Transcripts Per Million
VFDB	Virulence Factors Database
